# Local Hemodynamic Changes Immediately after Correction of an Aberrant Right Subclavian Artery in a Dog: A Contrast Computed Tomographic Study

**DOI:** 10.3390/vetsci8060104

**Published:** 2021-06-08

**Authors:** Yohei Mochizuki, Shoma Mikawa, Kenji Kutara, Keisuke Sugimoto, Hirosei Sakoya, Akihiro Ohnishi, Kanna Saeki, Yuki Shimizu, Teppei Kanda, Taketoshi Asanuma

**Affiliations:** 1 Faculty of Veterinary Medicine, Okayama University of Science, 1-3 Ikoinooka, Imabari, Ehime 794-8555, Japan; y-mochizuki@vet.ous.ac.jp (Y.M.); s-mikawa@vet.ous.ac.jp (S.M.); k-sugimoto@vet.ous.ac.jp (K.S.); a-oonishi@vet.ous.ac.jp (A.O.); ka-saeki@vet.ous.ac.jp (K.S.); y-shimizu@vet.ous.ac.jp (Y.S.); t-kanda@vet.ous.ac.jp (T.K.); t-asanuma@vet.ous.ac.jp (T.A.); 2 Sigenobu Animal Hospital, 1054-1 Ushibuchi, Toon, Ehime 791-0213, Japan; zeek1hs@hotmail.com

**Keywords:** aberrant right subclavian artery, contrast computed tomography, dog, hemodynamic change

## Abstract

A 1-year-old female Akita dog was referred for intermittent regurgitation. Computed tomographic angiography (CTA) showed an aberrant right subclavian artery (ARSA), resulting in constriction of the esophagus. After surgical ligation of the ARSA, CTA showed that the ARSA was not enhanced by contrast medium, and that sufficient collateral circulation of the right forelimb was supplied through the vertebral artery. Furthermore, the right and left vertebral arteries merged into the basilar artery at the level of the atlas, and no abnormal expansion of the ventral spinal artery was observed. Overall, we demonstrated the importance of post-surgical CTA for identification of surgical complications, including the formation of abnormal vessel alterations.

## 1. Introduction

The aberrant right subclavian artery (ARSA) is a rare form of vascular ring anomalies (VRAs) in dogs [1,2,3,4,5,6,7,8,9,10,11]. VRAs are congenital vascular defects resulting from abnormal aortic arch development in young dogs and cats [10,11]. Several types of VRAs have been reported in dogs, and the most common form is a persistent right aortic arch with a left arteriosum [1,2,3,4,5,6,7,8,9]. The other types include a double aortic arch, persistent right ductus arteriosus, and aberrant left subclavian artery. VRAs can lead to esophageal constriction and dilation. If an animal with a VRA presents with these clinical signs, surgical intervention may be indicated since medical treatment alone is likely to lead to a poor clinical outcome [6,11]. Two techniques have been described for the surgical treatment of ARSA, namely, (1) suture ligature and sectioning of the right subclavian artery, which is performed on dogs and humans, and (2) surgical revascularization, which is performed only on humans [6,11]. The existing literature in veterinary medicine states that the vertebral artery can provide sufficient collateral circulation after ligation of the ARSA [11]; however, to the best of our knowledge, no published report has described this hemodynamic change visually. This is the first report to demonstrate the hemodynamic changes after ligation of the ARSA by using computed tomographic angiography (CTA).

## 2. Case Presentation

**Figure 1:**
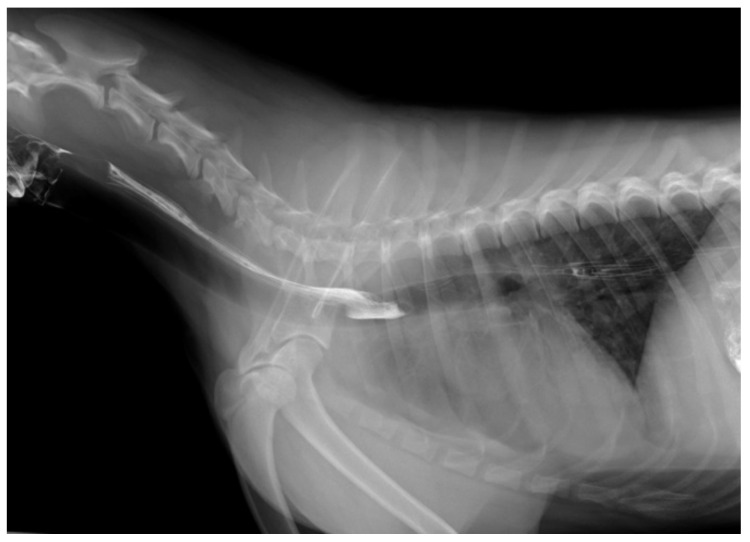
Pre-operative right lateral barium contrast study of the esophageal radiograph. The X-ray image revealed persistence of the contrast medium and mild esophageal dilatation at the level of the third intercostal space.

A 1-year-old female Akita dog weighing 22.1 kg was referred to Okayama University of Science Veterinary Medical Teaching Hospital (OUS-VMTH) with intermittent regurgitation and aspiration pneumonitis. No significant abnormalities were identified in the complete blood cell count or serum biochemical analysis. Thoracic radiographs from the referral hospital taken two months earlier revealed an alveolar pattern in the right pulmonary caudal lobe, which disappeared one month later. A barium contrast study of the esophagus revealed persistence of the contrast medium and mild esophageal dilatation at the level of the third intercostal space (Figure 1).

**Figure 2:**
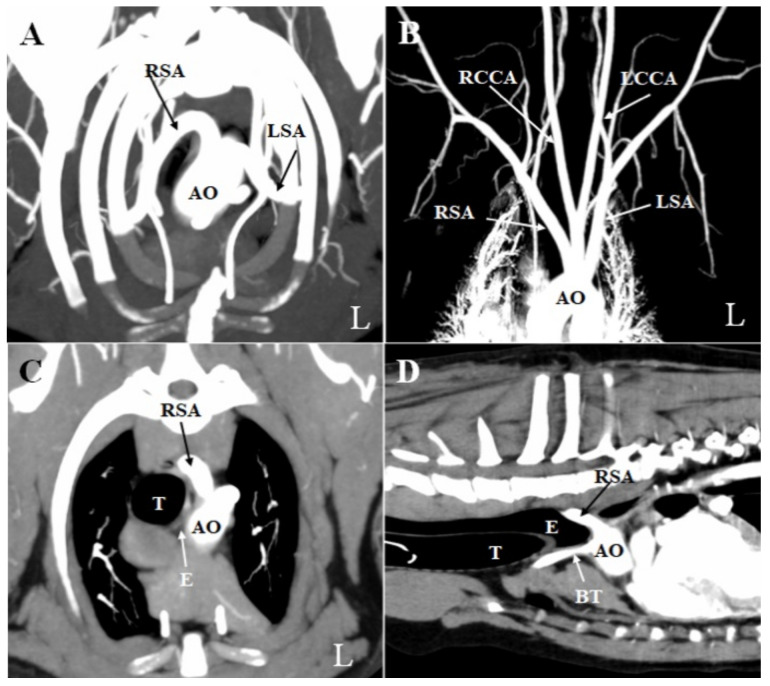
Pre-operative computed tomographic angiography images. (**A**) Multiplanar reconstruction of the transverse image. (**B**) Multiplanar reconstruction of the coronal image (bone deleted). An anomalous right subclavian artery that originated from the aortic arch at the same location as the left subclavian artery to the left of the trachea was identified. It then coursed cranially and obliquely toward the right forelimb, passing dorsally to the esophagus. (**C**) Transverse image at the level of the junction between the anomalous right subclavian artery and the aorta. The esophagus was constricted between the right subclavian vessel dorsally and bilaterally, and the trachea ventrally. (**D**) Sagittal image of the thorax. The esophagus located before the anomalous right subclavian artery was expanded. AO: aorta; BT: brachiocephalic trunk; E: esophagus; L: left; LCCA: left common carotid artery; LSA: left subclavian artery; RCCA: right common carotid artery; RSA: right subclavian artery; T: trachea.

No significant abnormalities were observed on the thoracic radiograph taken at OUS-VMTH. CTA and esophagoscopy were performed under general anesthesia. First, whole-body CTA was performed with a 16-slice multidetector scanner (Aquilion Lightning; Canon Medical Systems, Otawara, Japan) to obtain pre-contrast images. Next, intravenous iopamidol (2.5 mL/kg; Oypalomin 300, Fuji Pharma Co., Tokyo, Japan) was administered via the right cranial branch of the lateral saphenous vein to enhance the veins in the forelimb. The bolus-triggering technique was used for CTA. This technique was performed as follows: (1) A region of interest was selected in the descending aorta at the diaphragmatic level. (2) A series of low-dose, non-incremental scans were obtained, and the attenuation within the region of interest was constantly monitored. (3) The arterial-phase scan was acquired spontaneously when an enhancement of 150 HU was reached within the aorta. (4) A standard delay of 20 s was set between the arterial phase and the venous phase, and the delayed phase was acquired 2 min after administration of the contrast agent injection, per standard practice. CTA revealed an aberrant right subclavian artery (ARSA). This vessel originated from the aortic arch at the same location as the left subclavian artery, to the left of the trachea (Figure 2A,B). It then coursed cranially and obliquely toward the right forelimb, passing dorsally to the esophagus. The esophagus was constricted between the right subclavian vessel dorsally and bilaterally, and the trachea ventrally (Figure 2C). Furthermore, the portion of the esophagus located before the ARSA was dilated (Figure 2D).

**Figure 3:**
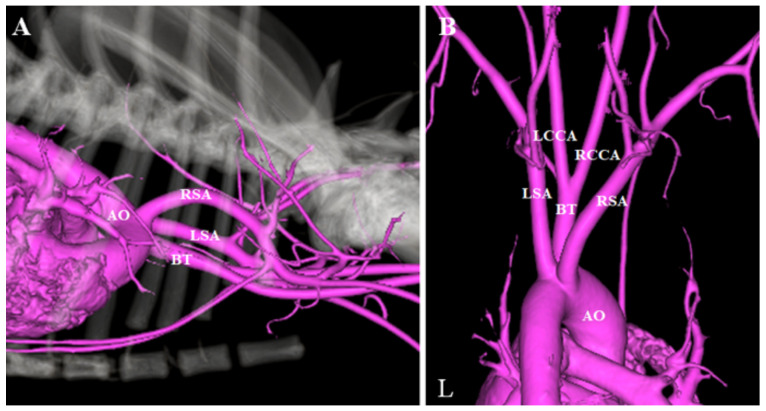
Three-dimensional reconstruction computed tomographic angiography images. (**A**) Right to left view. (**B**) Dorsoventral projection view. AO: aorta; BT: brachiocephalic trunk; L: left; LCCA: left common carotid artery; LSA: left subclavian artery; RCCA: right common carotid artery; RSA: right subclavian artery.

Three-dimensional reconstructions of the CTA images were generated to further illustrate the anatomy of the ARSA (Figure 3). Esophagoscopy confirmed extramural dorsal compression of the esophagus by an obliquely transverse pulsating tubular structure at the same level as the one observed in the thoracic radiograph. On the basis of these findings, we determined that the cause of intermittent vomiting was a compression of the esophagus by the ARSA.

After a discussion with the owner and obtaining informed consent, suture ligature and sectioning of the right subclavian artery were performed to relieve intermittent regurgitation. Preanesthetic medications included fentanyl (3 µg/kg IV), ketamine (0.5 mg/kg IV), and robenacoxib (2 mg/kg SC). The dog was anesthetized with propofol (6 mg/kg IV) and intubated. General anesthesia was maintained with sevoflurane mixed with 100% O_2_ and infusion of fentanyl (10 µg/kg/h) and ketamine (0.6 mg/kg/h) at a constant rate, under pressure-controlled ventilation. After intubation, an intercostal nerve block was performed on the incision area with 0.5% bupivacaine followed by administration of cefmetazole sodium (20 mg/kg IV). Heart rate, pulse rate, invasive arterial blood pressure, electrocardiography tracings, respiratory rate, tidal volume, minute volume, rectal temperature, arterial oxygen saturation, end-tidal carbon dioxide tension, and sevoflurane concentration were monitored continuously throughout the surgery. The patient was positioned in right lateral recumbency and a left-sided third intercostal thoracotomy was performed. The left cranial lung lobe was packed off caudally with a gauze square. Careful blunt dissection identified three vessels originating from the aortic arch. The ARSA was carefully isolated with right-angle forceps by bluntly dissecting around it. To determine if sufficient collateral circulation could be provided to the right thoracic limb, the ARSA was completely occluded using bulldog forceps, and blood flow was detected using a Doppler flow probe on the right paw. After confirming sufficient collateral circulation, the ARSA was ligated using a 3-0 Glycomer 631 (Biosyn, Covidien Japan, Tokyo, Japan) and transected.

**Figure 4:**
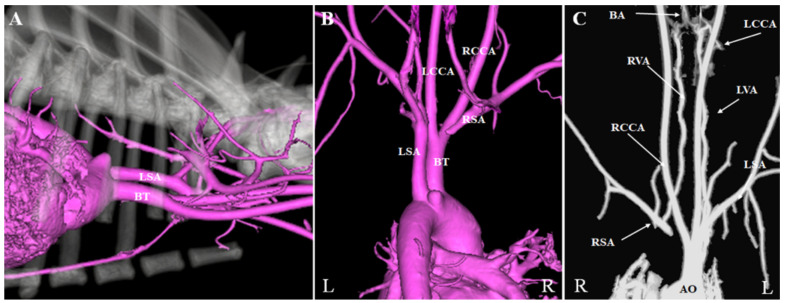
Three-dimensional reconstruction and multiplanar reconstruction of computed tomographic angiography performed after surgery. (**A**) Right to left view. (**B**) Dorsoventral projection views. (**C**) Multiplanar reconstruction of the coronal image (bone deleted). The ligated abnormal right subclavian artery was not enhanced. Circulation of the right forelimb was supplied via the vertebral artery. The right and left vertebral arteries merged to form the basilar artery at the level of the atlas. Abnormal expansion of the ventral spinal artery was not observed. AO: aorta; BA: basilar artery; BT: brachiocephalic trunk; L: left; LCCA: left common carotid artery; LSA: left subclavian artery; LVA: left vertebral artery; R: right; RCCA: right common carotid artery; RSA right subclavian artery; RVA: right vertebral artery.

A thoracostomy tube was placed through the 4th intercostal space, and the thorax was closed in a routine manner. Air was manually evacuated from the thoracic cavity. The patient received robenacoxib (40 mg PO SID) for 3 days after the surgery. A post-operative CTA revealed that the ligated ARSA was not enhanced by contrast (Figure 4A,B). Furthermore, circulation to the right forelimb was supplied via the vertebral artery (Figure 4C). The right and left vertebral arteries merged into the basilar artery at the level of the atlas. No abnormal expansion of the ventral spinal artery was observed.

The dog was fed a commercial comprehensive nutritional diet. A follow-up examination conducted one month later revealed that the dog was clinically normal, active, and exhibited no evidence of regurgitation. The general condition of the dog was still stable 140 days after the surgery.

## 3. Discussion

This is the first report demonstrating the use of CTA to depict the pre- and post-surgical anatomy of an ARSA. ARSA in dogs is rare, with only 17 cases described to date [1,2,3,4,5,6,7,8,9]. However, the previous surgical reports did not include visualization of collateral circulation of the right forelimb [6]. In the previous reports, diagnostic CTA was used for ARSA diagnosis [7,8,9]. However, in these cases, CTA after surgery was not performed. Further, in the present case, ligation of the abnormal right subclavian artery was performed without transection, since transection of the right subclavian artery may result in failure of adequate circulation to the right forelimb. The authors recommend measuring blood flow to the affected thoracic limb before and after occlusion to determine if sufficient collateral circulation is provided. In this report, blood flow was detected using a Doppler flow probe before and after ligation of the aberrant right subclavian artery to confirm sufficient collateral circulation. Furthermore, post-operative CTA established that the collateral circulation of the right forelimb was supplied via the vertebral artery. This finding is in accordance with similar results described in a textbook [11].

In the post-surgical CTA, the right and left vertebral arteries were seen to connect in the basilar artery at the level of the atlas, and there was no abnormal expansion of the ventral spinal artery. Furthermore, paralysis and hypotension of the right forelimb and neurological symptoms due to vertebrobasilar ischemia were not seen at all after surgery. In humans, constriction and obstruction of the subclavian artery may cause hypotension of the right upper limb [12,13] as well as neurological symptoms due to vertebrobasilar insufficiency [13,14], the so-called “subclavian steal syndrome” [13]. In veterinary medicine, intradural–extramedullary spinal cord compression associated with multilevel ectatic anastomotic radicular arterial branches connecting the left and right vertebral arteries was observed in patients showing a congenital lack of patency of the right subclavian artery [15]. Furthermore, stenosis and aneurysmal dilation of ARSA may cause hypotension of the right upper limb [16]. In the present case, no abnormalities were observed after surgery. However, abnormality caused by lack of patency of the right subclavian artery may occur after ligation of the ARSA, because this surgery changes the blood circulation in a similar way as that in a patient with multilevel ectatic anastomotic radicular arterial branches connecting the left and right vertebral arteries. Furthermore, the potential post-operative complications of decreased circulation of the right forelimb or no collateral circulation formation in the right forelimb cannot be ignored. Therefore, post-operative CTA is important to identify not only the collateral circulation of the right forelimb, but also the presence of any abnormal vessel alterations within the collateral circulation after surgery for ARSA. In conclusion, we used CTA to establish that collateral circulation of the right forelimb was supplied via the vertebral artery after ligation of an abnormal right subclavian artery. Post-surgical CTA of the ARSA was important to identify the presence of surgical complications, including the formation of abnormal vessel alterations.

## Data Availability

The data presented in this study are available in the manuscript.
